# A Higher Number of TMS-Elicited MEP from a Combined Hotspot Improves Intra- and Inter-Session Reliability of the Upper Limb Muscles in Healthy Individuals

**DOI:** 10.1371/journal.pone.0047582

**Published:** 2012-10-15

**Authors:** Andisheh Bastani, Shapour Jaberzadeh

**Affiliations:** Department of Physiotherapy, School of Primary Health Care, Faculty of Medicine, Nursing and Health Sciences, Monash University, Melbourne, Victoria, Australia; French National Centre for Scientific Research, France

## Abstract

We aimed to determine, using transcranial magnetic stimulation (TMS), the number of elicited motor evoked potentials (MEPs) that induces the highest intra- and inter-sessions reliability for the extensor carpi radialis (ECR) and first dorsal interosseus (FDI) muscles. Twelve healthy subjects participated in this study on two separate days. Single pulse magnetic stimuli were triggered with Magstim 200^2^ to obtain MEPs from the muscles of interest, with the subjects in a relaxed position. Reliability of MEP responses was investigated in three blocks of 5, 10 and 15 trials. The intra- and inter-session reliability of the MEPs' amplitudes and latencies were assessed using intraclass correlation coefficients (ICCs). Repeated measures ANOVA and paired t-tests revealed no significant time effect in the MEP amplitude and latency measurements (P>0.05). The ICCs indicated high intra-session reliability in the MEPs' amplitudes for the ECR and FDI muscles (0.77 to 0.99, 0.90 to 0.99, respectively) and latency (0.80 to 1.00, 0.75 to 0.97, respectively). The MEPs' amplitudes also had high inter-session reliability (0.84 to 0.97, 0.88 to 0.93, respectively), as did their latency (0.80 to 0.90, 0.75 to 0.97, respectively). Highest intra- and inter-session reliability was achieved for blocks of 10 and 15 trials. Our data suggest that intra- and inter-session comparisons should be performed using at least 10 MEPs in “combined hotspot” stimulation technique to ensure highest reliability.

## Introduction

Transcranial magnetic stimulation (TMS) is a non-invasive, safe and painless technique for assessment of brain corticospinal excitability in both healthy individuals and patients with neurological conditions [1]–[4]. One of the major advantages of TMS is the ability of the magnetic pulses to pass unchanged through the scalp to induce an electric current in underlying conductive brain tissues [1], [5], [6]. When applied over the primary motor cortex (M1) of a target muscle, TMS depolarizes nerve cells descending corticospinal pathways to contralateral muscle(s) of interest and elicits a motor response called “motor evoked potential” (MEP). This response can be recorded using surface electromyography (EMG) electrodes placed over the target muscle(s) [7]–[9].

*TMS-induced MEPs have been used* as a reliable outcome measure of corticospinal excitability changes *in a range of research protocols* [10]–[12]. Two important characteristics of recorded MEPs are amplitude and latency, which provide valuable information about corticospinal pathways. MEP amplitude is an indicator of M1 corticospinal excitability [13]: larger amplitudes indicate higher excitability and smaller amplitudes indicate lower excitability [13]. On the other hand, variation in MEP latency indicates change in the central and peripheral conduction time required for transmission of induced action potential from the M1 to the target muscle(s) [9].

A significant aspect of any clinical or experimental assessment tool is its test-retest reliability [7], [8], [14], [15]. Reliability refers to ‘the consistency of measurements’ [16]: it tests the stability of scores over time and involves the degree to which repeated measurements provide similar results [17]. A reliable measurement of MEPs guarantees stable amplitude size and latency in different testings over time [14], [18]. Reliability assures that any changes observed in the repeated measure designs and/or pre- and post-therapeutic interventions are genuine and are due to physiological changes rather than errors arising from methodological variabilities [19].

Previous studies suggested a relationship between the number of recorded MEPs and the level of reliability [14], [15], [20]. Studies using a mean of 5 recorded MEPs resulted in good to high reliability in amplitude measures compared to studies involving one to four MEPs per block; for example, Christie et al's recordings of two, three or four MEPs per block resulted in poor reliability [14], [20]. Recent intra- and inter-session reliability studies of MEPs also suggest that recorded MEPs are more reliable when larger numbers of trials are recorded and averaged for analysis [14], [15], [20]. Doeltgen et al. reported that an average of 10 MEPs provides high reliability in inter-session measurements [15]. The number of MEPs required to produce reliable measurement may vary in different settings and be specific to the study design, number of examinees, assessment and reliability measurement methods or techniques, and recorded muscle(s) of interest.

Despite the widespread use of TMS in recent years, few studies have focused on the test-retest reliability of resting MEPs in upper limb muscles [14], [20], [21]. Two studies, showed moderate reliability in MEP amplitude for the abductor digiti minimi (ADM) and first dorsal interosseus (FDI) in healthy individuals [14], [20]. In contrast, Livingstone et al. (2008) reported less consistency in the resting MEP amplitude for the abductor pollicis bravis (APB), FDI and ADM muscles. Nevertheless, Livingstone et al's [21] MEP amplitude coefficients were lower than those reported by Kamen [20] for the biceps muscle, prompting the hypothesis that the reliability of MEP amplitude may be muscle specific [20], [22]. In addition, Kamen [20] reliably measured MEP amplitude during simultaneous M1 stimulation of the biceps and FDI muscles (0.95 and 0.081, respectively). On the other hand, Livingston and Ingersoll [21] demonstrated high reliability for MEP latency obtained from APB, FDI and ADM muscles [21].

In TMS studies several researchers found a single hotspot for a given muscle and then analyzed MEPs simultaneously evoked from that site but in other muscles for which the TMS parameters were not optimized [7], [15], [20], [21], [23]. This is not a flawless approach and fails to show a complete picture of cortical changes in all targeted muscles. To address this issue, it might be better to use a “combined hotspot” with overlap M1s for all muscles of interest.

To our knowledge, while investigations of the intra- and inter-session MEP reliability of multiple upper limb muscles exist [7], [15], [20], [21], [23], no researchers have assessed the reliability of MEPs recorded from a “combined hotspot”, which could be useful for the studies in which MEPs of two or more muscles are simultaneously elicited.

The purpose of the current study was to compare the intra- and inter-session reliability of peak-to-peak amplitude and latency of different blocks of simultaneous elicitation of MEPs from the combined hotspot for ECR and FDI muscles at rest. We hypothesized that MEPs elicited from a combined hotspot, with optimized parameters for all target muscles, are reliable. Due to the stochastic nature and trial-to-trial variability of the TMS-elicited MEPs in all muscles and the fact that averaging may reduce this variability, we also hypothesized that there is a direct relationship between the number of MEPs in each block and reliability, and that the intraclass correlation coefficients (ICCs) of MEPs amplitude and latency are not muscle specific.

## Materials and Methods

### 2.1. Subjects

Twelve healthy volunteers (six women, six men) with a mean age of 30.3±6.8 (yrs) (range 21 to 47 yrs) a mean weight of 74.5±10.4 (kg) and a mean height of 171.4±7.8 (cm) were tested in two sessions separated by at least 48 hours. All were consistent right-handers according to the 10-item version of the Edinburgh Handedness Inventory (mean laterality index = 100) [24]. Prior to the experiments, all participants completed the Adult Safety Screening Questionnaire [25] to determine their suitability for TMS. Participants were informed about the experimental procedures and gave written informed consent according to the declaration of Helsinki. All experimental procedures were approved by the Monash University Human Research Ethics Committee. Each subject was tested at the same time of the day to avoid diurnal variation.

### 2.2. Electromyography (EMG) recording

Participants were seated in an adjustable podiatry chair, with the right forearm pronated and the wrist joint in neutral position on the arm rest. To ensure good surface contact and reduce skin resistance, a standard skin preparation procedure of cleaning and abrading was performed for each site of electrode placement [26]–[28]. MEPs were recorded from the right ECR and FDI muscles at rest, using pre-gelled self-adhesive bipolar Ag/AgCl disposable surfaces electrodes with an inter-electrode distance of 3 cm for the ECR and 2 cm for the FDI muscle (measured from the centres of the electrodes). The locations of ECR and FDI muscles were determined based on anatomical landmarks [29] and observations of muscle contraction in the testing position (wrist extension and radial deviation for ECR, and index finger abduction for FDI muscle) [30]. The accuracy of EMG electrode placement was verified by asking the subject to maximally contract the muscles of interest while the investigator monitored online EMG activity. The ground electrode was placed ipsilaterally on the styloid process of the ulnar bone [31]. Then, the electrodes were secured with tape. All raw EMG signals were band-pass filtered (10–500 Hz), amplified (×1000) and sampled at 1000 Hz and collected on a PC running commercially-available software (LabChart™ software, ADInstruments, Australia) via a laboratory analogue-digital interface (The PowerLab 8/30, ADInstruments, Australia) for later off-line analysis.

### 2.3. Measurement of corticospinal excitability by TMS

Participants were seated upright and comfortable with head and neck supported by a head rest. Single pulse magnetic stimuli were delivered using a Magstim 200^2^ (Magstim, UK) stimulator with a flat 70 mm figure-of-eight magnetic coil. Using the international 10–20 system, the vertex (C_z_) point was measured and marked to be used as a reference [28]. The magnetic coil was placed over the left hemisphere (cortex), contralateral to the target muscles. The coil was set at an angle 45° to the midline and tangential to the scalp, such that the induced current flowed in a posterior-anterior direction. To determine the optimal site of stimulation (hotspot), the coil was moved around the M1 of the target muscles to trigger the M1 overlapped area for both the ECR and FDI muscles that gave the largest MEP response. This overlapped M1 area was called the “combined hotspot”.

**Figure 1:**
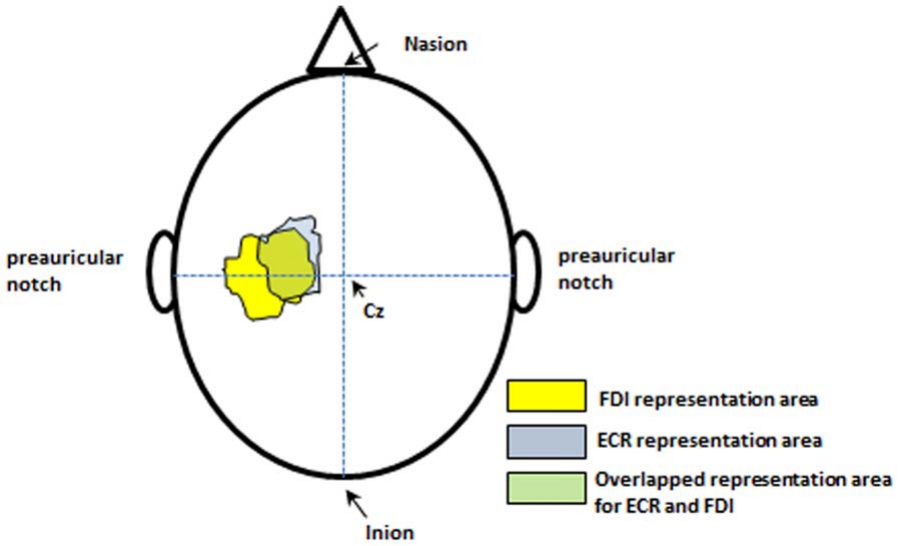
Contours plot of the ECR and FDI M1 representations. The overlap between the two representations is shown in green. Cz: The position of vertex. Adapted with modification from Devanne et al. (2006) study [32].

The surface area of representation and the coordinates of the combined hotspot for the FDI and ECR muscles were found and marked based on the size of the MEP amplitude. As illustrated by Devanne et al. [32], the optimal spot for stimulation of the FDI muscle is more anteriorly and laterally located relative to the vertex than that for the ECR muscle. Figure 1 explains the concept of the combined hotspot.

After localizing the optimal stimulation site, the coil position was marked on the scalp to ensure consistency in the placement of the coil for the remainder of the testing. The full hotspot identification procedure was performed in each session. Resting motor threshold (RMT) was defined as the minimal stimulus intensity that evoked 5 MEPs in a series of 10 tests with amplitude of at least 50 µV [*4]*, [6], [9], [33] from the combined hotspot of both ECR and FDI muscles. Hence, the same RMT was used for both muscles. The RMT for each subject was determined by increasing and decreasing stimulus intensity in 1–2% intervals until MEPs of appropriate size were elicited. For all further MEP measurement, the TMS intensity was set at 120% of each individual's RMT. Fifteen stimuli were elicited to assess corticospinal excitability at each time point. The stimulus intensity remained constant throughout the study session for each subject.

### 2.4. Procedures

All individuals participated in two experimental sessions. The protocol in session 1 enabled us to study the reliability of MEP amplitude and latency within a session (intra-session reliability). The corticospinal excitability of the ECR and FDI muscles was assessed at three consecutive time points (T1, T2 and T3) separated by intervals of 20 minutes. The EMG electrodes were left in place and the TMS coil was removed while the subjects rested for 20 minutes, with no hand or wrist movements allowed.

The second session of testing was held at least 48 hours after the first one. This session was shorter and only involved recording of MEPs at one time point (T1). Comparison of these data with the T1 from session 1 enabled us to study the inter-session reliability of MEPs' size and latency for the ECR and FDI muscles.

### 2.5. Data management and statistical analysis

Twelve subjects were required for a true *ρ* 0 of 0.7 against an alternative *ρ*1 of 0.9, based on a 95% significant level and a power of 80% (β = 0.20) for three time points [34].

The peak-to-peak amplitudes and latencies of elicited MEPs were measured for the ECR and FDI muscles. The MEPs' amplitudes were measured from the positive to the negative peak of the signals and MEPs latency was calculated from the stimulus artifact indicator to the first deflection of the signal. To assess the intra- and inter-session reliability of recorded MEPs, the averaged MEPs at each time point (T1, T2 and T3) were calculated in separate blocks of the first 5 (Block 1), first 10 (Block 2) and all 15 responses (Block 3).

Two-way repeated measure analysis of variance (ANOVA) and paired t-tests were used to detect systematic bias between the repeated measurements within or between days, respectively. This test shows the degree of agreement between the measurements and assesses the closeness of the repeated measures [35]. The correlation between the measurements was assessed using the ICC [8], which is the most appropriate reliability outcome measure for measurements on a continuous scale.

ICCs were calculated for blocks of the first 5, 10 and all 15 elicited MEPs in order to identify the number of trials which produced the greatest intra-session reliability. The same protocol was applied to calculate the inter-session reliability (between sessions 1 and 2) for MEPs' amplitudes and latencies. The ICCs, based on a two-way single measure mixed effects model (ICCs _(3,1)_), were calculated for averaged MEPs in each block for both inter- and intra-session reliability. The reliability coefficient ranges from 0 to 1, with values closer to 1 representing stronger reliability. Although the interpretation of ICCs is subjective, Portney and Watkins (2009) [35], suggested that coefficients below 0.50 represent poor reliability, from 0.50 to 0.75 correspond to moderate reliability, and values above 0.75 signal high reliability.

Unlike the ICC, which is a relative measure of reliability, standard error of measurement (SEM) was calculated which provides an absolute index of reliability [36]. The SEM quantifies the precision of individual scores on a test (within-subject reliability) and indicates to what extent the values observed at different time points vary from the ‘true’ value of that excitability parameter for a given subject [37]. The interpretation of SEM focuses on the assessment of reliability for individual subjects [38], and SEM determines the effect of measurement error on the test score of an individual examinee. SEM is estimated as follows: SEM = SD, where SD is the standard deviation of the scores from all subjects and ICC is the reliability coefficient [19], [37], [39]. The larger the SEM, the lower the reliability of the test and the less precision there is in the measurements taken and scores obtained.

All data are presented as mean±SD, the level of statistical significance was set at 5%, and all analyses were conducted using SPSS for Windows Version 19.

## Results

All participants completed both sessions of data collection. The mean interval between sessions of measurements was 52.7±4.6 hours.

### 3.1. Intra-session reliability

#### MEPs amplitude and latency

**Figure 2:**
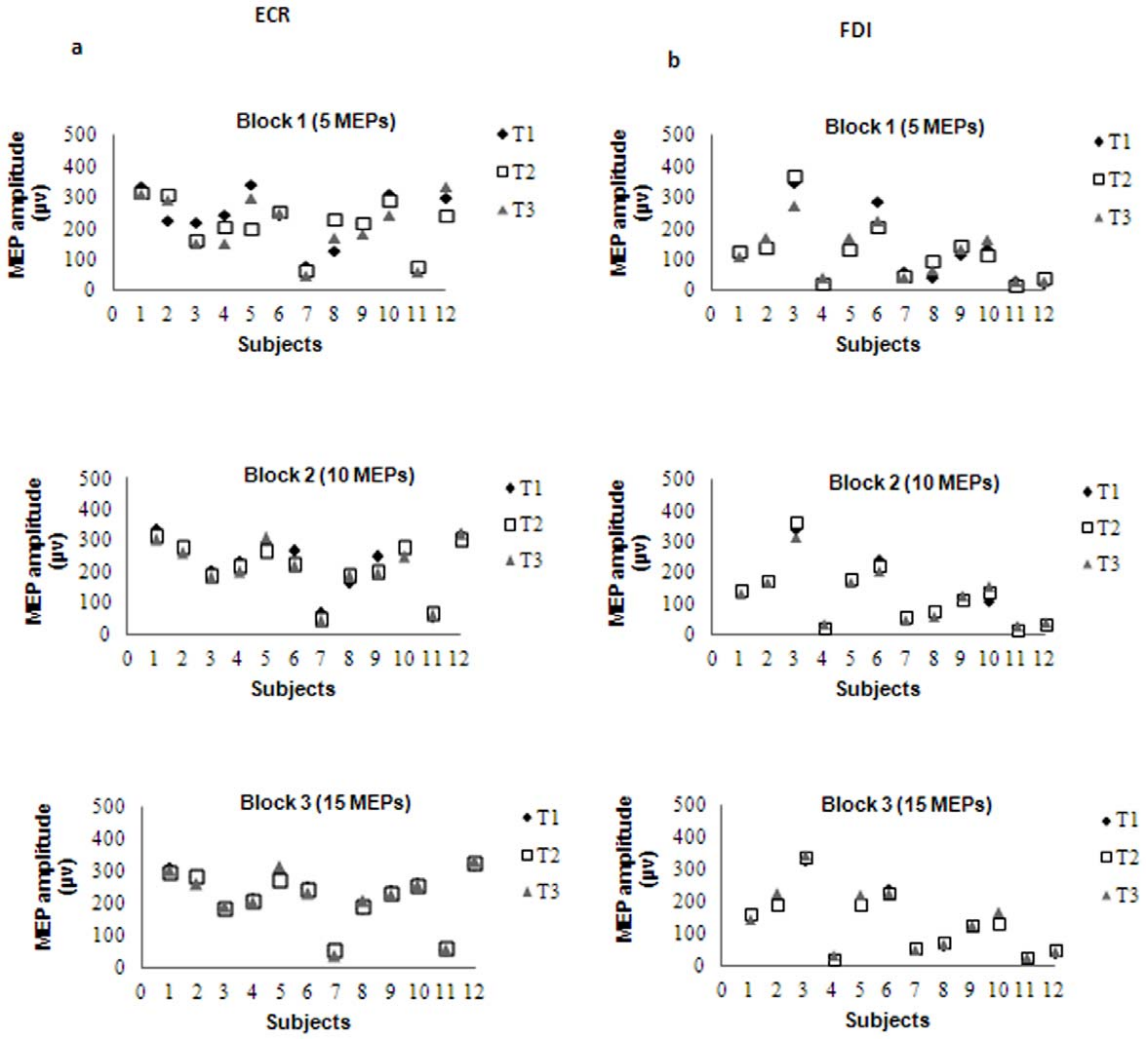
Comparison of MEPs amplitude in 12 subjects within a session. a) ECR, and b) FDI muscles with 5, 10 and 15 MEPs per block in three time points.

The averaged RMT and consequent stimulus intensity for both muscles were 45% (45.2±10.4) and 54% (54.3±12.5) of the stimulator output, respectively. A representative single subject's data (Figure 2a and b) showed minimal changes for the mean amplitude of the MEPs for ECR and FDI muscles at all three time points. Indeed, repetition of the measurements by the same examiner every 20 minutes after the first test revealed no significant differences in the group mean values of any of the measurements recorded (Tables 1 and 2). Repeated measures ANOVA revealed no significant time effect in any of the measurements for ECR muscle and FDI muscles (Tables 1 and 2).

**Table 1:** Mean, standard deviation and level of agreement of MEPs amplitude for three blocks of trials recorded from ECR and FDI muscles.

						ANOVA		Paired T-test	
		Mean amplitude (µv)±SD				(Intra session)		(Intra session)	
Muscle	Blocks	T1- session 1	T2- session 1	T3- session 1	T1- session 2	F (2,22)	P-value	T (11)	P-value
ECR	Block 1	229.51±95.63	215.92±80.27	208.99±95.98	221.07±78.95	0.97	0.39	0.59	0.56
	Block 2	228.34±90.64	215.94±85.06	212.23±88.89	217.13±83.71	0.73	0.44	1.49	0.16
	Block 3	224.0±88.04	220.91±85.45	220.81±0.92	215.8±83.66	0.59	0.55	1.70	0.11
FDI	Block 1	121.42±102.96	122.98±96.67	121.97±82.13	129.98±98.89	0.227	0.877	−0.60	0.56
	Block 2	124.70±95.76	132.17±100.17	124.99±85.94	132.74±99.40	0.571	0.638	−0.78	0.45
	Block 3	131.12±96.14	135.12±95.02	141.99±100.86	133.64±101.33	0.678	0.571	−0.24	0.81

**Table 2:** Mean, standard deviation and level of agreement of MEPs latency for three blocks of trials recorded from ECR and FDI muscles.

						ANOVA		Paired T-test		
		Mean latency (ms)±SD				(Intra session)		(Intra session)		
Muscle	Blocks	T1- session 1	T2- session 1	T3- session 1	T1- session 2	F (2,22)	P-value		T (11)	P-value
ECR	Block 1	16.66±1.07	16.50±1.24	16.58±1.24	16.41±0.99	0.47	0.62		1.39	0.19
	Block 2	16.66±1.30	16.58±0.99	16.58±1.37	16.75±0.96	0.18	0.83		−0.32	0.75
	Block 3	16.66±1.30	16.66±1.30	16.66±1.30	16.75±1.05	0.314	0.815		−0.56	0.58
FDI	Block 1	22.66±1.15	22.66±1.23	22.58±1.44	22.83±0.93	0.401	0.753		−0.69	0.50
	Block 2	22.41±1.08	22.91±1.24	22.58±1.16	22.75±1.21	1.486	0.236		−1.44	0.16
	Block 3	22.41±1.31	22.75±1.42	22.50±1.31	22.91±1.24	0.647	0.59		−2.17	0.05

ICCs ranged from 0.77 for block 1 (5 MEPs) to 0.99 for block 3 (15 MEPs). MEP amplitudes showed high reliability within a session for both ECR and FDI muscles (Table 3). As expected, higher ICCs were achieved for blocks of 10 and 15 MEPs in all comparisons.

**Table 3:** Comparison of between MEPs correlation of the recorded MEPs amplitude from ECR and FDI muscles.

		Intra session reliability					Inter session reliability	
		ICCs					Inter session reliability	
Muscle	Blocks	T1- T2	T1- T3	T2-T3	T1-T2-T3	SEM	T1	SEM
ECR	Block 1	0.77	0.90	0.82	0.83	12.70	0.84	8.93
	Block 2	0.97	0.96	0.97	0.97	2.62	0.95	3.37
	Block 3	**0.99**	0.98	0.98	0.98	1.15	**0.97**	1.62
FDI	Block 1	0.94	0.93	0.90	0.93	5.29	0.88	7.87
	Block 2	**0.99**	0.97	0.97	0.98	1.52	**0.93**	3.67
	Block 3	**0.99**	0.98	0.98	0.98	1.24	**0.93**	3.60

Largest ICCs values of each comparison are in bold. ECR: extensor carpi radialis; FDI: first dorsal interosseus; ICCs: inter class correlations; SEM: standard error of measurement.

The mean and ICC results for MEP latency of the ECR and FDI muscles are shown in Tables 2 and 4, respectively. MEP latency showed high stability over the three replicates within a session for both the ECR (ICCs = 0.80 to 1.00) and FDI (ICCs = 0.75 to 0.97) muscles. As expected, slightly higher ICCs were achieved for blocks of 10 and 15 trials in all comparisons.

**Table 4:** Comparison of between MEPs correlation of the recorded MEPs latency from ECR and FDI muscles.

		Intra session reliability					Inter session reliability	
		ICCs					ICCs	
Muscle	Blocks	T1- T2	T1- T3	T2-T3	T1-T2-T3	SEM	T1	SEM
ECR	Block 1	0.80	0.90	0.91	0.87	0.10	0.82	0.1
	Block 2	0.83	0.97	0.87	0.90	0.07	0.89	0.13
	Block 3	**1.00**	**1.00**	**1.00**	**1.00**	-	**0.90**	0.05
FDI	Block 1	0.75	0.86	0.87	0.83	0.14	0.75	2.18
	Block 2	0.76	0.94	0.88	0.89	0.13	0.77	2.06
	Block 3	0.88	**0.97**	0.89	0.91	0.05	**0.80**	1.97

Largest ICCs values of each comparison are in bold. ECR: extensor carpi radialis; FDI: first dorsal interosseus; ICCs: inter class correlations; SEM: standard error of measurement.

### 3.2. Inter-session reliability

#### MEP amplitude and latency

**Figure 3:**
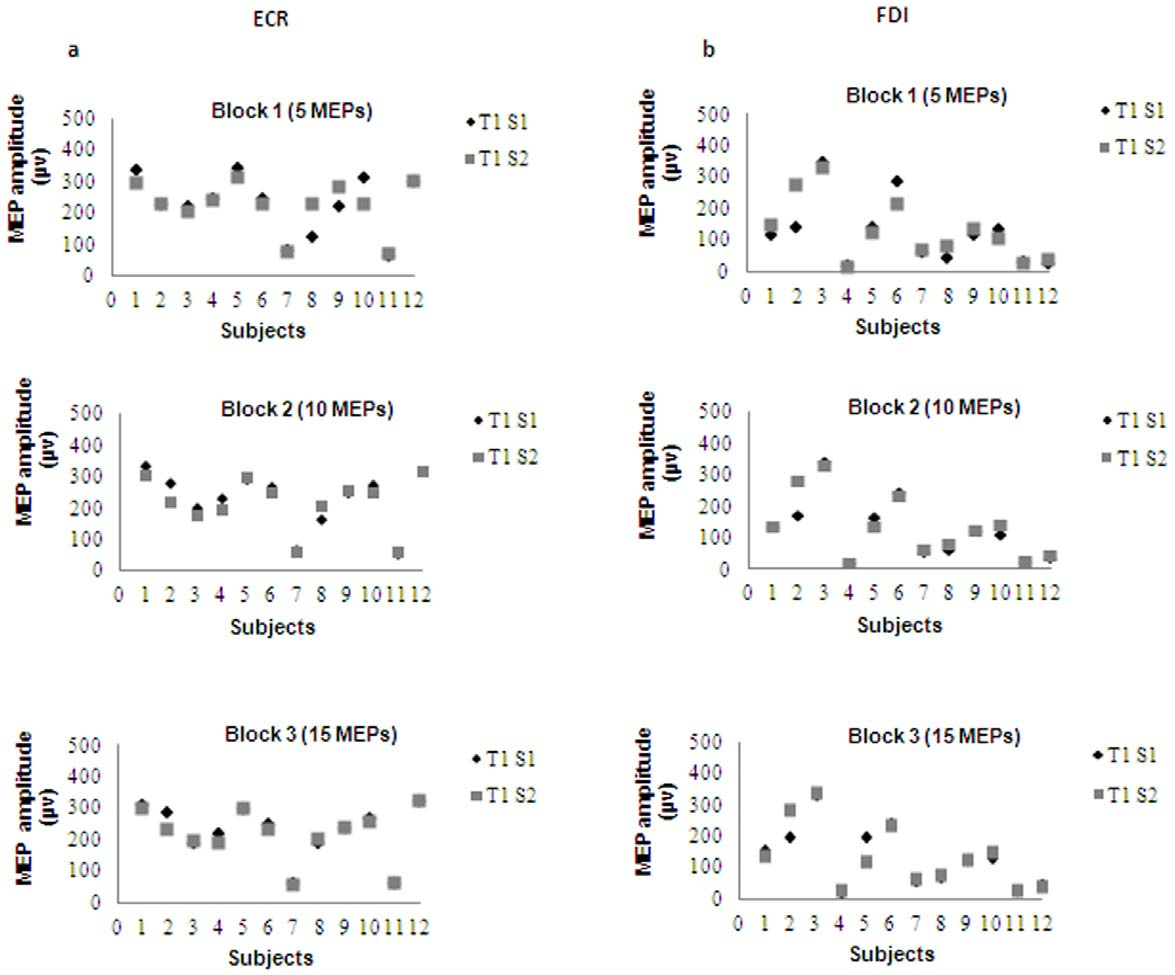
Comparison of MEPs amplitude in 12 subjects between two sessions. a) ECR and b) FDI muscle with 5, 10 and 15 MEPs per block in three time points.

The averaged RMTs and consequent stimulus intensities for both muscles were 46% (46±10.8) and 55% (55.2±13.0) of stimulator output, respectively. A representative single subject's data showed minimal changes in mean MEP amplitude for the ECR and FDI muscles (Figure 3a and b). Moreover, repetition of the measurements by the same examiner in two different sessions held at least 48 hours apart did not reveal any significant differences in the group mean MEP amplitude and latency values (Tables 1 and 2). Paired t-tests comparing the means of all variables between the two sessions showed no statistically significant differences for the ECR and FDI muscles (Tables 1 and 2). ICCs for MEP amplitudes ranged from 0.84 for block 1 (5 MEPs) to 0.97 for block 2 (10 MEPs) for the ECR muscle and 0.88 for block 1 (5 MEPs) to 0.93 for block 2 (10 MEPs) for the FDI muscle. Marginally higher ICCs were achieved for block 3 (15 MEPs) for the ECR muscle, with no change in the ICCs of the FDI muscle for blocks of 10 and 15 trials (Table 3).

ICC values for MEP latency ranged from 0.82 to 0.90 for block 1 and 2 (5 and 10 MEPs, respectively) for ECR and 0.75 to 0.80 for block 1 and 2 (5 and 10 MEPs, respectively) for the FDI muscle (Table 4). As expected, slightly higher ICCs were achieved for block 3 (15 MEPs) in all comparisons. The ICCs were higher in all three blocks for the ECR muscle compared to the FDI muscle.

## Discussion

In this study we assessed the intra- and inter-session reliability of the amplitude and latency of different blocks of simultaneous TMS-elicited MEPs from the ECR and FDI muscles. Correlations between individuals and sessions were determined using ICCs [8]. Systematic bias was evaluated by measuring the level of agreement using ANOVA or a paired t-test where appropriate. The reliability established in this study is also intra-rater reliability, because all data were collected by the same rater.

### 4.1. Intra-session reliability

The agreement and high values of ICCs between pre- and post-MEP measurements observed in both ECR and FDI muscles indicate high within-session reliability. These intra-session reliability results are in agreement with those of Christie et al. [14], who reported an ICC of 0.97 for the reliability of MEP amplitude derived from the ADM muscle. Furthermore, MEP latencies ranged from 16.4±0.9 ms for the ECR to 22.9±1.2 ms for the FDI muscles, results in agreement with MEP latency data reported by Ravnborg and Dahl [40] and Wu et al. [41]. As expected, motor evoked latencies demonstrated an absolute intra-session consistency for the FDI muscle and very high reliability for the ECR muscle. This can be explained by the careful positioning of EMG surface electrodes within the session and the consistency in the alignment and position of the TMS magnetic coil on the combined M1.

The results indicate a direct relationship between the number of recorded MEPs in each block of stimulation and the level of reliability, supporting the hypothesis of our study. We established high reliability in this session for 5, 10 and 15 MEPs per block, indicating that even an average of 5 MEP amplitudes is enough to establish high within-session reliability, in agreement with Christie et al. [14]. This result also supports Kamen's [20] findings of good to high reliability of MEP amplitude in the FDI and biceps muscles in healthy individuals.

### 4.2. Inter-session reliability

The agreement and also high and consistent ICCs indicate high inter-session reliability of MEP measurement in both ECR and FDI muscles. The ICCs of all three blocks in the present experiment are larger than those reported by Kamen [20] for the FDI muscle (0.60–0.81) and Christie et al. [14] for the ADM muscle (0.65–0.83). Although no previous reliability studies focused on forearm muscles, our ICCs for the ECR muscle were comparable with Kamen's findings [20] for the biceps muscle (0.95–0.99) for blocks of 10 and 15 MEPs. These values are higher than those reported by Livingston and Ingersoll [21], who found small (0.28) to moderate (0.72) ICCs for the FDI, APB and ADM muscles. Our results indicate that MEP amplitude remains constant in healthy subjects, even with a 48 hour interval between testing sessions.

MEP latency is sensitive to electrode positioning [42], particularly given that electrode placement over forearm muscles is inevitably more variable than in intrinsic hand muscles. Therefore, the high reliability of MEP latency found in this study suggests the consistent positioning of EMG electrodes across the two sessions. Although the reliability of MEP latency has not been previously investigated for forearm muscles, our results are in keeping with those of Livingston and Ingersoll [21], who showed that the MEP latency of distal hand muscles remained stable, with an ICC of 0.87 across different sessions.

In this study, the combined hotspot was more toward the periphery for the FDI muscle. Therefore, one potential explanation for the small MEPs recorded in the FDI muscle is that the MEP size might be smaller in the periphery of the cortical representation compared to that at the hotspot. However, it is interesting to see that the reliability remained high despite this small MEP size. In agreement with previous studies, reliability measures reached high values when 5 trials were included in the present analysis, with a slight increase in reliability when 10 or all 15 trials were considered. As the highest reliability was achieved by increasing the number of MEPs per block, we recommend the use of at least 10 MEP trials when the research includes multiple independent sessions of data collection and simultaneous M1 stimulations.

The high reliabilities demonstrated by high ICCs for MEP amplitude and latency in our study are in agreement with data reported for the upper limb muscles by some authors, regardless of whether they had used ICCs [7], [43], ANOVA or coefficient of variation (CV) [44], [45] for the statistical analysis. The ICC values recorded in the present study showed an overall reliability of over 0.75 in both the intra- and inter-session assessments.

The shape, size and orientation of the coil are main factors that determine the size of stimulated area as well as the direction of the induced current flow [46]. Moreover, a factor that could theoretically affect MEP amplitudes' reliability is the use of a neuronavigation system in eliciting MEPs. However, two recent studies found no decrease in the variability [47] and no further improve in reliability [48] of MEPs with TMS navigated systems. We used a conventional TMS assessment technique without a navigation system, but our results were in agreement with previous studies demonstrating high reliability in TMS mapping parameters with smaller numbers of MEPs, both with [49] and without [14] the use of a neuronavigation system.

The results support our hypothesis that TMS-elicited MEPs are not muscle specific. High reliability in both ECR and FDI muscles confirms data reported by Lefebvre et al. [50] demonstrating that TMS reliability is not muscle specific. However, Kamen [20] produced contradictory findings indicating that reliability varies according to the muscle of investigation, and that higher reliability in the biceps muscle could be a function of its location or M1 size in comparison to distal hand muscles.

It is important to note that SEM values were lower in blocks 2 and 3 (10 and 15 MEPs, respectively) than in block 1 (5 MEPs) for both the ECR and FDI muscles. In addition, SEM was similar in blocks 2 and 3 for the FDI muscle. Overall, the SEM became smaller as the number of MEPs per block increased from 5 to 15. As the observed values lie within the SEM from the true score, this shows the significance of increasing the number of recorded MEPs to bring the observed values closer to the true scores.

Based on the data presented here, TMS-elicited MEPs can be reproduced with a high degree of consistency to simultaneously assess the corticospinal pathways from both ECR and FDI muscles when performed in a controlled laboratory environment. Our findings are also useful for interpreting individual intervention effects in TMS-related studies where any changes in MEP responses can be considered as an intervention effect. TMS is frequently used in investigations such as brain mapping or recruitment curves, and can involve 250 or more stimulations. Our results indicate acceptable reliability with 5 MEPs per block, enabling researchers to avoid unnecessary stimulations to the brain. However, to increase the reliability of inherently variable and sensitive measurements, more MEPs per block should be recorded.

One limitation of our study is that we studied only healthy young participants, so findings cannot be extrapolated to older and/or unwell subjects. This study was also limited in that it only evaluated one intensity (120% RMT), so we are unable to expand our findings to higher or lower intensities, although previous studies have shown that stimulation by higher intensities provides higher reliability [49].

The results of our study only indicate intra-rater reliability. An obvious further direction is to perform similar study by testing the inter-rater reliability for multi-center studies.
