# Correction: A Higher Number of TMS-Elicited MEP from a Combined Hotspot Improves Intra- and Inter-Session Reliability of the Upper Limb Muscles in Healthy Individuals

**DOI:** 10.1371/annotation/ca8c5e69-bdd5-4ac2-9c42-993792c37b33

**Published:** 2013-09-11

**Authors:** Andisheh Bastani, Shapour Jaberzadeh

In the second from last sentence of the fifth paragraph of subsection "2.5. Data management and statistical analysis," there was an error in the in-line equation.

The sentence should read, "SEM is estimated as follows: SEM = SD√1-ICC, where SD is the standard deviation of the scores from all subjects and ICC is the reliability coefficient [19, 37, 39]." 

